# Chitosan-Based Carbon Dots with Applied Aspects: New Frontiers of International Interest in a Material of Marine Origin

**DOI:** 10.3390/md20120782

**Published:** 2022-12-16

**Authors:** Angel M. Villalba-Rodríguez, Reyna Berenice González-González, Manuel Martínez-Ruiz, Elda A. Flores-Contreras, María Fernanda Cárdenas-Alcaide, Hafiz M. N. Iqbal, Roberto Parra-Saldívar

**Affiliations:** 1 School of Engineering and Sciences, Tecnologico de Monterrey, Monterrey 64849, Mexico; 2 Institute of Advanced Materials for Sustainable Manufacturing, Tecnologico de Monterrey, Monterrey 64849, Mexico

**Keywords:** nanomaterials, waste valorization, N-doped carbon dots, hydrothermal synthesis, microwave, polysaccharide

## Abstract

Carbon dots (CDs) have attracted significant research attention worldwide due to their unique properties and advantageous attributes, such as superior optical properties, biocompatibility, easy surface functionalization, and more. Moreover, biomass-derived CDs have attracted much attention because of their additional advantages related to more environmentally friendly and lower-cost synthesis. In this respect, chitosan has been recently explored for the preparation of CDs, which in comparison to other natural precursors exhibited additional advantages. Beyond the benefits related to the eco-friendly and abundant nature of chitosan, using it as a nanomaterial precursor offers additional benefits in terms of structure, morphology, and dopant elements. Furthermore, the high content of nitrogen in chitosan allows it to be used as a single carbon and nitrogen precursor for the preparation of N-doped CDs, significantly improving their fluorescent properties and, therefore, their performances. This review addresses the most recent advances in chitosan-based CDs with a special focus on synthesis methods, enhanced properties, and their applications in different fields, including biomedicine, the environment, and food packaging. Finally, this work also addresses the key challenges to be overcome to propose future perspectives and research to unlock their great potential for practical applications.

## 1. Introduction

Carbon dots (CDs) have attracted extensive attention due to their unique properties in UV fluorescence and their outstanding performances in uses such as cell-labeling materials and heavy metal sensors. CDs are described as zero-dimensional carbon nanoparticles with a particle size below 10 nm, quasi-spherical shaped, and optically active [1,2]. Furthermore, CDs stand out among most nanomaterials due to their unique properties. These are: biocompatibility, photoluminescence, chemical stability, enzyme mimicking, surface functionality, hydrophilicity, and simple synthesis methods, among others.

The development and understanding of CDs have significantly increased in recent times due to the research efforts of the scientific community, reflected by the increasing number of publications and growing interest in this type of nanomaterial [3]. Concurrently, their potential applications have been extended to different areas. For example, much literature has been published on the employment of CDs to detect toxic elements [4,5], remove emerging pollutants from water bodies [3], prepare energy storage devices [6], and act as artificial nanozymes for different catalytic applications [7], among other innovative applications. However, their synthesis through sustainable, cost-effective, and eco-friendly protocols remains challenging, as hydrothermal and microwave methods are the most common approaches [8,9]. Accordingly, natural, renewable, and economical carbon precursors play a crucial role since their utilization might increase the viability and competitiveness of the process providing sustainable alternatives and economical materials for the large-scale production of CDs [10]. In this manner, an inexpensive material can be converted into a precious product with great potential for biomedical and biotechnological applications. Recent studies have explored the synthesis of CDs from different natural and waste materials, including chitosan, an amino polysaccharide recovered from marine waste with remarkable advantages as a carbon precursor. Chitosan is extracted from chitin, an abundant nitrogenous biopolymer found in crustaceans or insects. It is the second most abundant natural polymer after cellulose; approximately 1010–1011 t of chitin are recovered annually and produced into chitosan [11,12].

**Figure 1:**
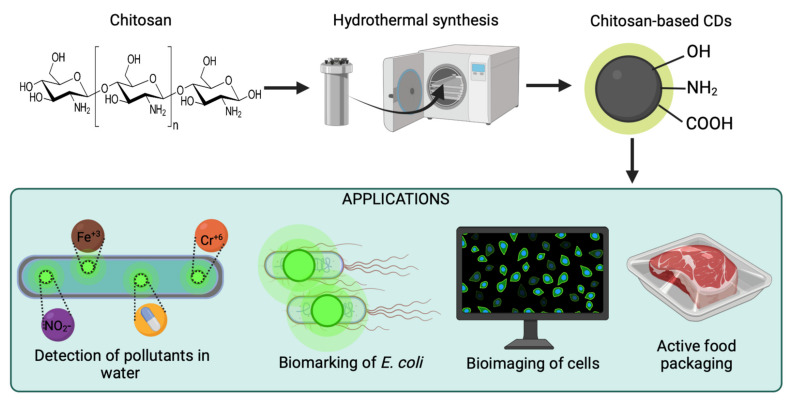
Schematic representation of the synthesis of chitosan-based carbon dots for applications such as the detection of pollutants, active food packaging, biosensing, and bioimaging. Created with BioRender.com and extracted under premium membership.

In addition to its abundant, economical, and renewable nature, chitosan has additional benefits. For instance, it possesses high amounts of nitrogen [13], and multiple functional groups, including acetamido, amino, and hydroxyl groups [12]. This is highly relevant since chitosan can act as single carbon and nitrogen precursor to easily obtain N-doped carbon nanomaterials, which are advantageous for multiple applications [13]. The outstanding properties of chitosan have been documented in different studies reporting the synthesis of chitosan-based materials, including carbon spheres, porous carbon, nanoparticles, and, more recently, CDs [14,15,16,17]. A few reviews provide a broader perspective of carbon materials derived from chitosan [12,18,19]; however, none summarizes the most recent advances in chitosan-based CDs. Figure 1 shows a schematic representation of the preparation of chitosan-based CDs for several applications. This review addresses the structure and properties of chitosan-based CDs, their synthesis methods, and their applications in different fields, including biomedicine, the environment, and energy. Finally, current challenges are identified to propose further research directions to reach practical applicability.

## 2. Chitosan as a Carbon Source for Nanomaterials

**Figure 2:**
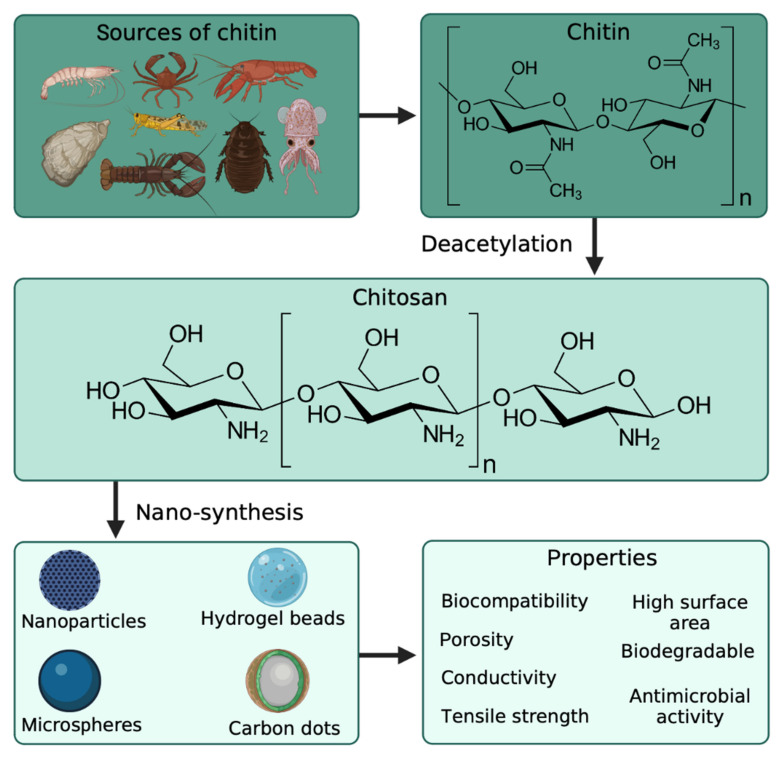
Sources of chitosan for the preparation of chitosan-based nanomaterials with enhanced properties. Created with BioRender.com and extracted under premium membership.

Chitin is a polymer derived from the exoskeleton of arthropods and mainly recovered for commercialization from shrimp, crabs, and other crustaceans (Figure 2). It is a linear polysaccharide built from beta-1,4 linked N-acetylglucosamine units, and it is highly insoluble in common solvents [20]. Chitosan is the biopolymer obtained from the deacetylation of chitin, and it can also be seen as a copolymer of d-glucosamine N-acetylglucosamine. Compared to chitin, chitosan is soluble in an acidic medium such as water acidified with acetic acid, with only low concentrations needed (commonly 1% *v*/*v* acetic acid is used). Its biocompatibility, biodegradability, and gel-forming properties make it easy to handle and manage as a polymeric matrix. However, chitin and chitosan have been used in tissue engineering for different applications, such as wound healing and cellular growth [21]. Chitin has shown highly hydrophobic properties caused by the N-acetylglucosamine polymeric structure, making it a rigid material. Still, it also has good electric properties when modified with maleic anhydride or reinforced with carbon nanotubes. This may be viable in biomedical applications requiring electrical conductivity [22]. These properties can be used for engineering scaffolds for neuron growth, nerves, and the treatment of neurodegenerative diseases [23]. Additionally, no reports of anti-inflammatory or allergic responses were observed in human subjects’ ingestion, injection, implantation, and topical application [24]. Amongst other important properties of chitosan, its degradation rate is influenced by the degree of deacetylation (d-glucosamine/N-acetylglucosamine ratio), which may be an essential factor for different biomedical and biotechnological applications [25].

The chemical structure of chitosan helps achieve cell adhesion and proliferation properties. Additionally, due to the highly hydrophilic surface it has, it promotes cell growth [24]. In addition, chitosan has shown antibacterial properties and can generate scaffolds with high porosity [26]. Although it has many advantages due to its properties, chitosan has poor mechanical strength, making it challenging to maintain a predefined shape for transplantation. However, this has been improved by cross-linking and copolymerizing this biomaterial with other polymers. Some attempts focused on reinforcement with nanoparticles [27], or incorporating hydroxyl-apatite, beta-tricalcium phosphate, alginate, and gelatin as copolymers. The cross-linkage of these polymers may result in better scaffolds that allow the construction of high mechanical strength tissues. In this manner, chitosan has prepared different nanomaterials, providing them with superior chemical, mechanical, and physical properties, including tensile strength, porosity, surface area, electrical conductivity, and thermal stability. The outstanding properties of chitosan-based nanoformulations have shown great potential in different research areas, such as drug delivery, bioimaging, sensing, gene delivery, diagnosis, and treatment of several diseases [28,29]. Different nanoformulations derived from chitosan can be found; however, the most commonly prepared are chitosan nanoparticles. Chitosan nanoparticles present low toxicity when studied in vivo and in vitro. Thus, it has been employed for biomedicine applications. Moreover, the surface of chitosan-based nanoparticles is typically positively charged, which, combined with their mucoadhesive properties, can adhere to mucus membranes for a sustained release of drugs [29].

Another attractive property of chitosan-based nanoparticles is their antibacterial activity. Thus, antimicrobial wound dressing applications have also been explored [30]. For instance, Babaee et al. [31] evaluated the effect of adding chitosan nanoparticles into a plasticized starch film for active packaging applications. Chitosan particles acted as a reinforcing and antimicrobial agent. Chitosan-based films presented excellent antibacterial activity against Gram-positive and Gram-negative bacteria; they exhibited inhibition zones against *S. aureus* and *E. coli* with a complete reduction (100%) of *S. aureus*. Moreover, mechanical and barrier properties were enhanced at higher concentrations (4 wt.%) of chitosan nanoparticles [31]. Similarly, some research groups have enhanced antimicrobial activity by incorporating antibiotics, metallic particles, or other compounds with antimicrobial effects. For example, Yeamsuksawat et al. [32] prepared and characterized Fe_3_O_4_@Chitosan@Ag nanoparticles. Nanoparticles showed superior antibacterial performance against *E. coli* and *S. cerevisiae*, exhibiting a 100% inhibition rate. The authors concluded that the synthesized nanomaterial could be used in different applications, including food packaging, biological, textile, and medical applications [32]. Similar developments have been reported in which chitosan has played a key role in the antimicrobial performance of various hybrid materials for applications such as inhibiting bacterial growth in a food or preserving paper documents [33,34].

Chitosan nanospheres have also been recently developed. As a representative example, Zhao et al. [35] prepared nanospheres from N,N,N-Trimethyl chitosan to encapsulate hydrophobic curcumin and thus overcome the tissue barriers due to the positive charge of the surface. It reported an excellent encapsulation efficiency, higher than 90%, with no toxic effect on cells. Interestingly, at higher concentrations of chitosan, an extended release of curcumin was observed; therefore, adjusting the concentration of chitosan could represent a suitable strategy to achieve an extended release of drugs [35]. 

Among the carbon-based nanomaterials, CDs are outstanding due to their multiple advantageous features, such as chemical stability, biocompatibility, easy surface functionalization, fluorescence, and economical synthesis. In order to propose more sustainable synthesis protocols, natural and waste precursors have been explored to prepare such novel nanomaterials [2,4]. In this respect, the carbon atoms in the glucosamine chains of chitosan serve as a source for the preparation of CDs for applications in the biomedical and environmental fields [36,37].

## 3. Methods for the Synthesis of Chitosan-Based Carbon Dots

Chitosan is an excellent precursor for preparing carbon nanoparticles, as it is an abundant and inexpensive renewable biomass source with high nitrogen content [12,38]. In the literature can be found many studies of CDs prepared through different processes and using a vast variety of precursors, in which citric acid stands out as a popular selection [39,40]. However, citric acid-based CDs usually require the simultaneous addition of nitrogen-rich co-precursors such as urea, ethylenediamine, and phenylalanine, among others, to provide nitrogen elements to the CDs’ structures and thus enhance their overall properties and performances [41,42,43]. 

In this context, the employment of chitosan as a precursor for CDs represents additional advantages since it is a single carbon and nitrogen source; thus, chitosan provides amino functional groups in addition to hydroxyl groups on the CDs’ surface. Several methods of chitosan-based CDs are summarized in Table 1 [44,45,46,47,48,49,50,51,52,53,54,55,56,57,58,59,60,61,62,63,64,65,66,67,68,69,70], for different applications in the biomedical and environmental fields. Generally, the presence of nitrogen elements on the CDs’ surface is highly desired, since nitrogen plays a fundamental role in maximizing the quantum yield [38]. In this manner, chitosan-based CDs present self-doped nitrogen elements that increase the surface-state defects resulting in enhanced optical and electronic properties, including photoluminescence and tailored electron properties due to the strong electron-donating capacity of nitrogen [17]. The most common functional groups on the surface of chitosan-based CDs include -NH_2_, -OH, and COOH [12,64].

**Figure 3:**
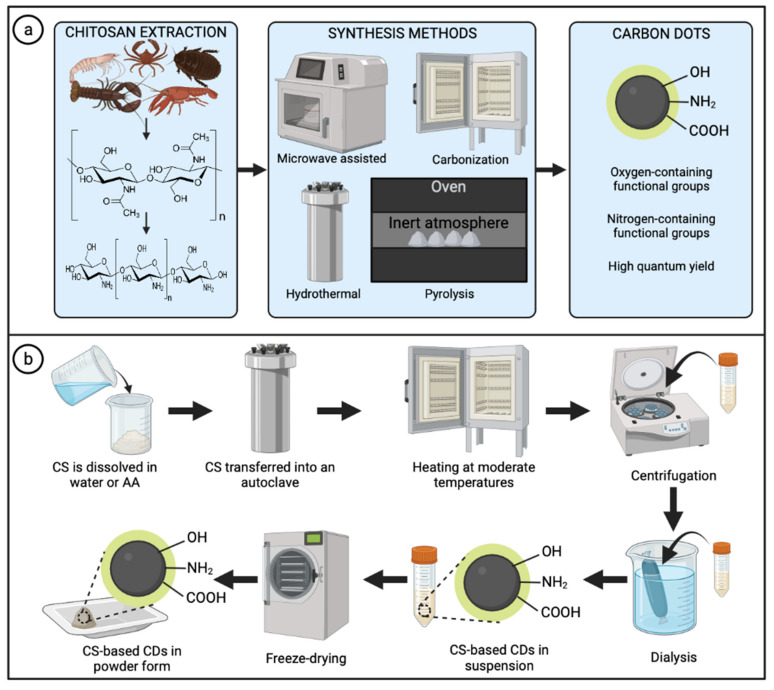
Synthesis methods for the preparation of chitosan-based carbon dots. (**a**) Chitosan extraction and most common synthesis methods to obtain carbon dots. (**b**) Typical methodology for the preparation of chitosan-based carbon dots through the hydrothermal method. Abbreviations: CS (chitosan); AA (acetic acid); CDs (carbon dots). Created with BioRender.com and extracted under premium membership.

Chitosan has been widely used to prepare novel CDs with excellent properties and great potential in different applications. The transformation of chitosan into such CDs can be achieved through different synthetic procedures, including hydrothermal, microwave-assisted, and pyrolysis, among others (Figure 3a). The relevance of the synthesis method is widely demonstrated in the literature; preparation methods have considerable effects on the electronic and optical properties of the CDs. For instance, Zattar et al. [38] compared chitosan CDs prepared by two methods: hydrothermal and acid dehydration. Interestingly, several differences were reported, including particle size distribution, nitrogen content, and quantum yield. Regarding particle size distribution, hydrothermally prepared CDs presented an average diameter of 2.4 nm. In comparison, CDs from acid dehydration showed a smaller average size of 1.5 nm with a narrower distribution attributed to the stronger acid attack during that method. This difference in diameter can be impactful depending on the type of application, as long as there is a consistent size average, which indicates a good synthesis protocol. Moreover, CDs prepared by the hydrothermal method exhibited a greater content of nitrogen-containing functional groups and, thus, a higher quantum yield (9.3%) than CDs prepared by acid dehydration with a quantum yield of 3.3% [38]. In this manner, synthesis methods directly impact the properties of the obtained CDs and their further applications.

The hydrothermal method is considered one of the most applied methods to obtain CDs due to its low cost, eco-friendliness, and facile control of parameters such as temperature and time of reaction [71,72,73,74]. Chitosan–alone or combined with other co-precursors–is typically dissolved in water or acetic acid solution and transferred into a Teflon-lined stainless-steel autoclave. Subsequently, the stainless-steel reactor must be adequately sealed and heated at moderate temperatures (below 220 °C) under autogenous pressure. After reactions occur and room temperature is reached, the obtained product is collected and separated/purified. Researchers have reported different purification methods, including filtration, centrifugation, and dialysis, among others, or a combination of some [64,75]. Then, CDs in solution are stored in refrigeration or freeze-dried to obtain solid samples for further applications or to meet requirements for some characterization techniques (Figure 3b) [76].

In this context, chitosan-based CDs reported by Ni et al. [17] were prepared from an ultrasonically dispersed suspension of chitosan in water, which was transferred into an autoclave and heated at 200 °C for 10 h. After room temperature was reached, the collected solution was centrifuged and filtered to remove larger particles. Finally, the supernatant was dialyzed and freeze-dried to obtain CDs in powder form [17].

A similar approach was followed by Xu et al. [77]; however, they used chitosan in combination with p-phenylenediamine as the source of carbon and nitrogen, which were dispersed in acetic acid. Moreover, higher temperatures and longer reaction times were used (220 °C for 18 h); in contrast, purification required only centrifugation and filtration to finally obtain CDs in suspension [77].

Hydrothermal synthesis is a very convenient method for preparing CDs derived from chitosan since the mild operating conditions in combination with water and the composition of the precursor lead to CDs with oxygen-containing and nitrogen-containing functional groups. Moreover, some molecules can be produced during the decomposition of the chitosan, such as CH_3_COOH and NH_3_, which persist in the reaction medium and cause the addition of heteroatoms into the structure of CDs through different chemical reactions. The introduction of heteroatoms in the structure of CDs represents significant improvements in quantum yield; in addition, the presence of nitrogen- and oxygen-containing surface functional groups improve the solubility and photoluminescence of the hydrothermally synthesized CDs [38]. 

The hydrothermally synthesized CDs derived from chitosan are obtained due to different transformation pathways; however, they might present significant variations in their properties, particle size, structure, and yields. The notable variability is due to the many factors involved, such as operational conditions (e.g., temperature, reaction time, heating rate), composition, properties, and structures of precursors. Further efforts should be directed to fully understand the formation mechanisms to optimize CDs’ yields and properties. Other methods have been employed for preparing chitosan-based CDs, such as the microwave-assisted method. This approach uses chitosan under microwave irradiation, receiving homogeneous heating directly to the target molecules. This is a notable difference since other synthesis methods heat the precursors convectively or conductively. One of the main advantages of this method is the reaction time, which is significantly shorter compared to the hydrothermal methods, which require a longer reaction time, usually around 5 h to 24 h. For instance, Pawar et al. [53] prepared passivated CDs using chitosan gel as the carbon precursor and poly(ethylene glycol) as the passivating agent. Their methodology consisted of microwave heating at 600 W at 100 °C within 3 min. Their CDs presented a particle size of 2.3–7.6 nm and a quantum yield of 25%, showing great potential for detecting trace amounts of water in organic solvents [53]. However, this synthesis method has challenges, such as low synthetic yield. In this manner, Liu et al. [78] proposed a microwave-hydrothermal carbonization method to reduce the reaction time and increase the synthesis yield; chitosan-based CDs were prepared within 30 min of the reaction with a high synthetic yield of 45.9%. Their method has the advantage of better controlling parameters, including temperature and pressure, which avoids side reactions and improves the overall synthetic yield. Moreover, CDs exhibited a quantum yield of 12.17% and the capacity to sensitively detect Fe^2+^ [78].

Pyrolysis, as a classical and simple thermal method, has also been employed for the preparation of CDs, including chitosan-based ones. This approach converts the carbon precursor into CDs via degradation and carbonization under an inert atmosphere and high temperatures. Typically, carbon precursors are placed inside the reactor, which is heated at a certain temperature with a controlled heating rate. The inert atmosphere is usually achieved by the introduction of nitrogen or argon gas. Inert gases should be introduced before heating to remove the oxygen in the system and avoid the combustion of the precursors. The main advantage of the pyrolysis method relies on its simplicity; however, it might require high temperatures and the obtained CDs can present broad particle size distribution and low values of quantum yield [73,79].

In addition, some properties of the CDs obtained by this method can be tuned by modifying the parameters of the process, such as the temperature, reaction time, heating rate, etc. [74]. As a representative example, Horo et al. [80] prepared CDs from low molecular weight chitosan and silk-fibroin blends under an inert atmosphere by introducing nitrogen flow. The pyrolysis was performed at 230 °C for 3 h. Then, a fine powder product was obtained after grinding, which was later dissolved in water. After filtration, CD samples were freeze-dried and stored for further analyses and applications [80]. Similarly, other research groups have applied pyrolysis under an inert atmosphere to prepare chitosan-based CDs [81,82]. The tailored photoluminescent properties of chitosan-based CDs prepared by the hydrothermal method have led researchers to employ them mainly for the sensitive and selective detection of several emerging pollutants in water samples (Table 2) [17,52,68,72,75,77,83,84,85,86,87].

## 4. Applications

### 4.1. Biomedicine

Being zero-dimensional (0D), quantum dots (QDs) are a nanomaterial from the carbon family. QDs have shown promising biomedical applications due to their size on the nanoscale. Carbon quantum dots are particularly useful in applications of the biomedical field, such as bioimaging, cell labeling, and biosensing. Chitosan-based CDs were applied for imaging human umbilical vein endothelial cells (HUVAC), which were exposed to low concentrations (50 µg/mL) of water-dispersed CDs. These CDs were synthesized through a simple one-pot hydrothermal method from a mixture of gum tragacanth and chitosan, which showed good detection of the cells by fluorescence microscopy due to the luminescence of the CDs intracellularly [45].

The same hydrothermal approach was used for synthesizing CDs from chitosan and applied for the bioimaging of A549 epithelial cells, which showed low cytotoxicity and good biocompatibility [58]. While CDs are commonly synthesized using the hydrothermal autoclave, another technique often used is pyrolysis through microwaves. The common one-pot method has the dissolved chitosan put under a microwave for several minutes until carbonized, as explained in the previous section. This technique has been used to synthesize CDs implemented in cellular labeling, bioimaging, and drug delivery [64,88]. Not only are the biocompatibility and non-toxicity of CDs properties relevant for the application of this nanomaterial in the biomedical field for bioimaging, biosensing, and drug delivery, but also the surface area to volume ratio which allows these materials to enter the extracellular matrix. The most important aspect is the photoluminescence, which provides for tunability of the color reflected by such material and the functionalization of the surface, depending on what type of application will be given to the final product.

### 4.2. Environmental

In terms of environmental applications of chitosan-based CDs, there have been many studies showing the capability of such nanomaterial for the detection of heavy metals in water such as Fe (III), Cu (II), and Cr (VI) [49,60,76], and some other specific applications as antibacterial agents in food packaging [72], detection of nitrite and enrofloxacin in water [76,81], algal growth enhancement, etc., [47]. In a recent study done by Wu et al. [71] CDs were obtained from the carbonization of chitosan and doped with nitrogen and phosphorus, using phosphoric acid as a medium. The carbon quantum dots showed luminescence from blue to green and orange, allowing for the tunability of the colors emitted from the material at different wavelengths depending on the type of application required and the desired emission. It was shown through this study that the addition of phosphoric acid as a solvent allowed for faster reaction times in the synthesis process, which, in this case, was a parallel hydrothermal microwave reaction.

The application scope of CDs in visible-light photocatalysis at longer wavelengths can be extended through this approach while allowing multi-color-emissive. The synthesis reaction can be shortened to only 5 min, and the fluorescence of the nanomaterial from blue to green to orange can be tailored by adjusting the acid concentrations. This can open up new possibilities in environmental and biotechnological applications for detecting microorganisms, heavy metal ions, microalgal growth, and many more [71].

### 4.3. Food Packaging

Antimicrobial properties are an important aspect of coating materials for applications such as food packaging to increase the shelf life of foods. With the help of nanotechnology, nanoparticles and nanocomposites can be synthesized to open a broader scope of novel options to tackle this problem in the global market. Thus, incorporating carbon quantum dots and other nanoparticles into these materials, particularly those obtained from natural sources such as chitosan, have been demonstrated to have antibacterial properties. These are mainly attributed to its polycationic nature, which interacts with the negatively charged surface of bacteria, altering cell permeability [89].

The incorporation of chitosan-based carbon quantum dots on carboxymethyl cellulose films for food packaging applications was performed by Riahi et al. [72], with average sizes around 7.2 nm, demonstrated not only an improvement in the mechanical properties of the film, but also a strong antibacterial effect on *E. coli* and *L. monocytogenes*, as well as considerable antioxidant activity, and antifungal activity on *A. niger* and *P. chrysogenum*. Additionally, the films showed almost null cytotoxicity against L929 cells at concentrations up to 500 μg/mL. Furthermore, mechanically, the addition of the chitosan-based quantum dots increased the tensile strength of the carboxymethyl cellulose film by up to 27.6% and its elastic modulus by approximately 61.5% [72]. Another approach is not using chitosan directly as the carbon source for quantum dots, but instead using it as the polymeric matrix in the form of thin films or fibers reinforced with CDs from other sources, such as kelp, allowing the CDs to do their antibacterial activity combined with that of the chitosan-based matrix and their respective biocompatibility/biodegradability properties [90]. In this context, CDs are good candidates for antibacterial and antifungal functions, whether using chitosan as the carbon source or as the polymeric matrix.

## 5. Current Challenges and Recommendations

Research on CDs has shown great progress in recent times, thus providing useful information and data for a better understanding of CDs’ properties, structures, and applications. However, there are still many key issues to be addressed. For instance, many synthesis methods have been reported for preparing chitosan-based CDs; however, scalable synthesis methodologies to produce high-quality CDs at high production yields remain unreported. In addition, the unclear effects of parameters, reaction conditions, and characteristics of the initial precursor make it difficult to control precisely the properties obtained in the CDs. In this respect, further efforts should be directed to understand the formation mechanisms in detail to get higher yields of CDs with controllable and enhanced properties.

Chitosan-based CDs have been employed for multiple applications, such as detecting toxic elements, active food packaging, and nanozymes for catalytic applications. Despite the efforts and progress in such applications, there is still investigation work to be done to achieve practical application in real scenarios. For example, the precise exploration of the role of CDs in each reaction and their interaction with other components or media is highly required. In addition, further examination of their toxicity and metabolic pathways on different organisms is extremely relevant for some applications such as biomedicine.

## 6. Conclusions

Chitosan-based CDs have demonstrated great potential in many research fields, including biomedicine, the environment, and energy. They possess enhanced fluorescence and optical properties owing to their precursor’s characteristics and benefits due to the eco-friendly and abundant nature of chitosan. In this review, we have discussed the enhanced properties of chitosan-based CDs directly associated with the precursor. Moreover, we have analyzed the most recent applications, highlighting the detection of different pollutants in environmental samples as the most common. However, other novel applications have been reported, such as the fabrication of active food packaging and their use as nanozymes. Current challenges that need to be addressed and some key insights regarding synthesis protocols, properties, and applications of chitosan-based CDs for future guidance have also been identified. The optimized synthesis methods capable of producing chitosan-based CDs on a large scale with precise control of their properties will considerably expand the spectrum of applications for chitosan-based CDs.

## Figures and Tables

**Table 1:** Overview of chitosan-based carbon dots: synthesis methods and applications.

Medium	Method	Application	Analyte	Reference
Water	Hydrothermal	Water remediation	Heavy metal ions	[44]
Water	Hydrothermal	Imaging	Endothelial cells	[45]
Epoxy resin	Hydrothermal	WLED; 3D Printing	None	[46]
Water	Hydrothermal	Algal growth	Astaxanthine	[47]
Ethanol	Hydrothermal	Bioimaging	A549 Cells	[48]
Water	Hydrothermal	Biosensing	Cr(VI) and H_2_O_2_	[49]
Agarose Hydrogel	Microwave	Water remediation	Heavy metal ions	[50]
Dry	Hydrothermal	Drug detection	Isoniazid	[51]
Water	Hydrothermal	Imaging	Nitrite; *E. coli* and *B. subtilis*	[52]
Water	Microwave	Detection	4-(pyridine-2-yl)-3H-pyrrolo[2,3-c]quinoline (PPQ)	[53]
Dansyl chloride	Hydrothermal	Sensing	Heavy metal ions	[54]
Hydrogen peroxide	Chemical reaction	Biosensing	*S*. *aureus*	[55]
Cadmium sulfide	Chemical reaction	Drug delivery	Sesamol	[56]
Water	Hydrothermal	Detection	Iodine ions	[57]
Water	Hydrothermal	Bioimaging	A549 cells	[58]
Ethanol	Hydrothermal	Detection	Heavy metal ions	[59]
Water	Hydrothermal	Sensing	Cu^2+^	[60]
Water	Hydrothermal	Remediation	CO_2_	[61]
Water and alcohol	Hydrothermal	Imaging and sensing	Electron transfer	[62]
Glassy carbon electrode	Hydrothermal	Corrosion inhibitor	BIS 2062 carbon steel	[63]
Water	Microwave	Cell labeling and drug delivery	Human dermal fibroblasts	[64]
Dry	Microwave	Cellular imaging	Human liver cancer HepG 2 cells	[65]
Water	Hydrothermal	Detection	ClO^−^	[66]
Dry	Microwave	Labeling and biosensors	None tested	[67]
Water	Microwave	Bioimaging	*S. aureus*	[68]
Water	Hydrothermal	Detection	Fe^3+^	[69]
Photocross-linked chitosan matrix	Microwave	Tissue engineering	None	[70]

**Table 2:** Overview of hydrothermally prepared carbon dots derived from chitosan: synthesis, properties, and applications.

CDs’ Type	Precursors	Hydrothermal Parameters	PS (nm)	QY (%)	Application	Reference
CDs	Chitosan and urea	190 °C, 15 h	7.1	16.81	Detection of Cr (VI) in water	[75]
CDs	Chitosan and p-phenylenediamine	220 °C, 18 h	3	19	Detection of nitrite and enrofloxacin in water	[77]
CQDs	Chitosan	180 °C, 12 h	7.8	NR	Active food packaging	[72]
Fe-CDs	Chitosan, citric acid, FeSO_4_.7H_2_O and ethylenediamine	180 °C, 6 h	15.69	28.09	Peroxidase-like nanozyme for managing bacterial biofilms fouling in environmental protection and food safety	[83]
CDs	Chitosan	200 °C, 10 h	2.13	38	Detection of trace water in organic solvents	[17]
N-CQDs	Chitosan	180 °C, 24 h	2	NR	Detection of Fe^3+^ in water	[68]
N,S-CDs	Chitosan and κ-carrageenan	220 °C, 18 h	8	59.31	Detection of Fe^3+^ and ascorbic acid	[84]
CDs	Chitosan	180 °C, 5 h	2.36–3.65	18.9	Ratiometric fluorescent determination of pH and enzyme reactions	[85]
CDs	Chitosan and tartaric acid	200 °C, 5 h	20	5.2	Detection of Fe^3+^ and ascorbic acid	[86]
N-CDs	Chitosan	180 °C, 12 h	2.8	35	Detection of nitrite and bacteria imaging	[52]
CDs	Chitosan	180 °C, 12 h	29.4	NR	Direct reduction of Ag^+^ to Ag^0^	[87]

Abbreviations: PS (Particle size); QY (quantum yield); CDs (Carbon dots); Fe-CDs (iron-doped carbon dots); N-CQDs (nitrogen doped-carbon quantum dots); N,S-CDs (nitrogen and sulfur co-doped carbon dots); NR (not reported).

## Data Availability

Not applicable.

## References

[1] Das S., Ngashangva L., Goswami P. (2021). Carbon Dots: An Emerging Smart Material for Analytical Applications. Micromachines.

[2] González-González R.B., González L.T., Madou M., Leyva-Porras C., Martinez-Chapa S.O., Mendoza A. (2022). Synthesis, Purification, and Characterization of Carbon Dots from Non-Activated and Activated Pyrolytic Carbon Black. Nanomaterials.

[3] González-González R.B., Sharma A., Parra-Saldívar R., Ramirez-Mendoza R.A., Bilal M., Iqbal H.M.N. (2022). Decontamination of Emerging Pharmaceutical Pollutants Using Carbon-Dots as Robust Materials. J. Hazard. Mater..

[4] González-González R.B., Parra-Saldívar R., Ramirez-Mendoza R.A., Iqbal H.M.N. (2022). Carbon Dots as a New Fluorescent Nanomaterial with Switchable Sensing Potential and Its Sustainable Deployment for Metal Sensing Applications. Mater. Lett..

[5] González-González R.B., Morales-Murillo M.B., Martínez-Prado M.A., Melchor-Martínez E.M., Ahmed I., Bilal M., Parra-Saldívar R., Iqbal H.M.N. (2022). Carbon Dots-Based Nanomaterials for Fluorescent Sensing of Toxic Elements in Environmental Samples: Strategies for Enhanced Performance. Chemosphere.

[6] Kumar S., Goswami M., Singh N., Sathish N., Reddy M.V., Kumar S. (2022). Exploring Carbon Quantum Dots as an Aqueous Electrolyte for Energy Storage Devices. J. Energy Storage.

[7] Lopez-Cantu D.O., González-González R.B., Melchor-Martínez E.M., Martínez S.A.H., Araújo R.G., Parra-Arroyo L., Sosa-Hernández J.E., Parra-Saldívar R., Iqbal H.M.N. (2022). Enzyme-Mimicking Capacities of Carbon-Dots Nanozymes: Properties, Catalytic Mechanism, and Applications—A Review. Int. J. Biol. Macromol..

[8] Hoang V.C., Hassan M., Gomes V.G. (2018). Coal Derived Carbon Nanomaterials–Recent Advances in Synthesis and Applications. Appl. Mater. Today.

[9] González-González R.B., González L.T., Iglesias-González S., González-González E., Martinez-Chapa S.O., Madou M., Alvarez M.M., Mendoza A. (2020). Characterization of Chemically Activated Pyrolytic Carbon Black Derived from Waste Tires as a Candidate for Nanomaterial Precursor. Nanomaterials.

[10] Kumar R., Singh R.K., Singh D.P. (2016). Natural and Waste Hydrocarbon Precursors for the Synthesis of Carbon Based Nanomaterials: Graphene and CNTs. Renew. Sustain. Energy Rev..

[11] Silvestre J., Delattre C., Michaud P., de Baynast H. (2021). Optimization of Chitosan Properties with the Aim of a Water Resistant Adhesive Development. Polymers.

[12] Hammi N., Chen S., Dumeignil F., Royer S., el Kadib A. (2020). Chitosan as a Sustainable Precursor for Nitrogen-Containing Carbon Nanomaterials: Synthesis and Uses. Mater. Today Sustain..

[13] Khan A., Goepel M., Colmenares J.C., Gläser R. (2020). Chitosan-Based N-Doped Carbon Materials for Electrocatalytic and Photocatalytic Applications. ACS Sustain. Chem. Eng..

[14] Guo L., Ding Y., Qin C., Song W., Sun S., Fang K., Li W., Du J., Wang F. (2018). Anchoring Mn_3_O_4_ Nanoparticles onto Nitrogen-Doped Porous Carbon Spheres Derived from Carboxymethyl Chitosan as Superior Anodes for Lithium-Ion Batteries. J. Alloys Compd..

[15] Narasimhamurthy K., Udayashankar A.C., de Britto S., Lavanya S.N., Abdelrahman M., Soumya K., Shetty H.S., Srinivas C., Jogaiah S. (2022). Chitosan and Chitosan-Derived Nanoparticles Modulate Enhanced Immune Response in Tomato against Bacterial Wilt Disease. Int. J. Biol. Macromol..

[16] Xi Y., Xiao Z., Lv H., Sun H., Zhai S., An Q. (2023). Construction of CuO/Cu-Nanoflowers Loaded on Chitosan-Derived Porous Carbon for High Energy Density Supercapacitors. J. Colloid. Interface Sci..

[17] Ni Y., Zhou P., Jiang Q., Zhang Q., Huang X., Jing Y. (2022). Room-Temperature Phosphorescence Based on Chitosan Carbon Dots for Trace Water Detection in Organic Solvents and Anti-Counterfeiting Application. Dye. Pigment..

[18] Ababneh H., Hameed B.H. (2021). Chitosan-Derived Hydrothermally Carbonized Materials and Its Applications: A Review of Recent Literature. Int. J. Biol. Macromol..

[19] Wang J., Zhuang S. (2022). Chitosan-Based Materials: Preparation, Modification and Application. J. Clean. Prod..

[20] Villalba-Rodriguez A.M., Parra-Saldivar R., Ahmed I., Karthik K., Malik Y.S., Dhama K., Iqbal H.M.N. (2017). Bio-Inspired Biomaterials and Their Drug Delivery Perspectives—A Review. Curr. Drug Metab..

[21] Kertmen A., Dziedzic I., Ehrlich H. (2022). Patentology of chitinous biomaterials. Part II: Chitosan. Carbohydr. Polym..

[22] Uzun I., Celik O. (2015). Physicochemical Characterization and the Comparison of Chitin and Chitin Modified with Maleic Anhydride. Orient. J. Chem..

[23] Singh N., Chen J., Koziol K.K., Hallam K.R., Janas D., Patil A.J., Strachan A., Hanley J.G., Rahatekar S.S. (2016). Chitin and Carbon Nanotube Composites as Biocompatible Scaffolds for Neuron Growth. Nanoscale.

[24] Thandapani G., Supriya Prasad P., Sudha P.N., Sukumaran A. (2017). Size Optimization and in Vitro Biocompatibility Studies of Chitosan Nanoparticles. Int. J. Biol. Macromol..

[25] Gámiz-González M.A., Correia D.M., Lanceros-Mendez S., Sencadas V., Gómez Ribelles J.L., Vidaurre A. (2017). Kinetic Study of Thermal Degradation of Chitosan as a Function of Deacetylation Degree. Carbohydr. Polym..

[26] Tao F., Cheng Y., Shi X., Zheng H., Du Y., Xiang W., Deng H. (2020). Applications of Chitin and Chitosan Nanofibers in Bone Regenerative Engineering. Carbohydr. Polym..

[27] Roy S., van Hai L., Kim H.C., Zhai L., Kim J. (2020). Preparation and Characterization of Synthetic Melanin-like Nanoparticles Reinforced Chitosan Nanocomposite Films. Carbohydr. Polym..

[28] Shukla S.K., Mishra A.K., Arotiba O.A., Mamba B.B. (2013). Chitosan-Based Nanomaterials: A State-of-the-Art Review. Int. J. Biol. Macromol..

[29] Mohammed M.A., Syeda J.T.M., Wasan K.M., Wasan E.K. (2017). An Overview of Chitosan Nanoparticles and Its Application in Non-Parenteral Drug Delivery. Pharmaceutics.

[30] Kravanja G., Primožič M., Knez Ž., Leitgeb M. (2019). Chitosan-Based (Nano)Materials for Novel Biomedical Applications. Molecules.

[31] Babaee M., Garavand F., Rehman A., Jafarazadeh S., Amini E., Cacciotti I. (2022). Biodegradability, Physical, Mechanical and Antimicrobial Attributes of Starch Nanocomposites Containing Chitosan Nanoparticles. Int. J. Biol. Macromol..

[32] Yeamsuksawat T., Zhao H., Liang J. (2021). Characterization and Antimicrobial Performance of Magnetic Fe_3_O_4_@Chitosan@Ag Nanoparticles Synthesized via Suspension Technique. Mater. Today Commun..

[33] Egil A.C., Ozdemir B., Gunduz S.K., Altıkatoglu-Yapaoz M., Budama-Kilinc Y., Mostafavi E. (2022). Chitosan/Calcium Nanoparticles as Advanced Antimicrobial Coating for Paper Documents. Int. J. Biol. Macromol..

[34] Duarte L.G.R., Picone C.S.F. (2022). Antimicrobial Activity of Lactoferrin-Chitosan-Gellan Nanoparticles and Their Influence on Strawberry Preservation. Food Res. Int..

[35] Zhao A., She J., Manoj D., Wang T., Sun Y., Zhang Y., Xiao F. (2020). Functionalized Graphene Fiber Modified by Dual Nanoenzyme: Towards High-Performance Flexible Nanohybrid Microelectrode for Electrochemical Sensing in Live Cancer Cells. Sens. Actuators B Chem..

[36] Iravani S., Varma R.S. (2020). Green Synthesis, Biomedical and Biotechnological Applications of Carbon and Graphene Quantum Dots. A Review. Environ. Chem. Lett..

[37] Bedian L., Villalba-Rodríguez A.M., Hernández-Vargas G., Parra-Saldivar R., Iqbal H.M.N. (2017). Bio-Based Materials with Novel Characteristics for Tissue Engineering Applications—A Review. Int. J. Biol. Macromol..

[38] Zattar A.P.P., Fajardo G.L., de Mesquita J.P., Pereira F.V. (2021). Luminescent Carbon Dots Obtained from Chitosan: A Comparison between Different Methods to Enhance the Quantum Yield. Fuller. Nanotub. Carbon Nanostructures.

[39] Ludmerczki R., Mura S., Carbonaro C.M., Mandity I.M., Carraro M., Senes N., Garroni S., Granozzi G., Calvillo L., Marras S. (2019). Carbon Dots from Citric Acid and Its Intermediates Formed by Thermal Decomposition. Chem.-A Eur. J..

[40] Mintz K.J., Zhou Y., Leblanc R.M. (2019). Recent Development of Carbon Quantum Dots Regarding Their Optical Properties, Photoluminescence Mechanism, and Core Structure. Nanoscale.

[41] Feng Z., Li Z., Zhang X., Shi Y., Zhou N. (2017). Nitrogen-Doped Carbon Quantum Dots as Fluorescent Probes for Sensitive and Selective Detection of Nitrite. Molecules.

[42] Chahal S., Yousefi N., Tufenkji N. (2020). Green Synthesis of High Quantum Yield Carbon Dots from Phenylalanine and Citric Acid: Role of Stoichiometry and Nitrogen Doping. ACS Sustain. Chem. Eng..

[43] Stachowska J.D., Murphy A., Mellor C., Fernandes D., Gibbons E.N., Krysmann M.J., Kelarakis A., Burgaz E., Moore J., Yeates S.G. (2021). A Rich Gallery of Carbon Dots Based Photoluminescent Suspensions and Powders Derived by Citric Acid/Urea. Sci. Rep..

[44] Wang P., Li L., Pang X., Zhang Y., Zhang Y., Dong W.F., Yan R. (2021). Chitosan-Based Carbon Nanoparticles as a Heavy Metal Indicator and for Wastewater Treatment. RSC Adv..

[45] Moradi S., Sadrjavadi K., Farhadian N., Hosseinzadeh L., Shahlaei M. (2018). Easy Synthesis, Characterization and Cell Cytotoxicity of Green Nano Carbon Dots Using Hydrothermal Carbonization of Gum Tragacanth and Chitosan Bio-Polymers for Bioimaging. J. Mol. Liq..

[46] Ni J., Huang X., Bai Y., Zhao B., Han Y., Han S., Xu T., Si C., Zhang C. (2022). Resistance to Aggregation-Caused Quenching: Chitosan-Based Solid Carbon Dots for White Light-Emitting Diode and 3D Printing. Adv. Compos. Hybrid Mater..

[47] Nguyen M.K., Park D., Lee Y.-C. (2021). Influence of Chitosan-Based Carbon Dots on Astaxanthin Production of Green Alga *Tetraselmis* sp.. J. Nanosci. Nanotechnol..

[48] Dutta P., Ghosh T., Kumar H., Jain T., Singh Y. (2015). Hydrothermal and Solvothermal Synthesis of Carbon Dots from Chitosan-Ethanol System. Asian Chitin J..

[49] Zhang Y., Dong Y., Zheng H., Yang X., Yao C. (2021). High Quantum Yield Fluorescent Chitosan-Based Carbon Dots for the Turn-On-Off-On Detection of Cr(VI) and H_2_O_2_. Nano.

[50] Gogoi N., Barooah M., Majumdar G., Chowdhury D. (2015). Carbon Dots Rooted Agarose Hydrogel Hybrid Platform for Optical Detection and Separation of Heavy Metal Ions. ACS Appl. Mater. Interfaces.

[51] Shekarbeygi Z., Farhadian N., Ansari M., Shahlaei M., Moradi S. (2020). An Innovative Green Sensing Strategy Based on Cu-Doped Tragacanth/Chitosan Nano Carbon Dots for Isoniazid Detection. Spectrochim. Acta Part A Mol. Biomol. Spectrosc..

[52] Sun L., Zhang H., Wang Y., Xiong Z., Zhao X., Xia Y. (2021). Chitosan-Derived N-Doped Carbon Dots for Fluorescent Determination of Nitrite and Bacteria Imaging. Spectrochim. Acta Part A Mol. Biomol. Spectrosc..

[53] Pawar S., Togiti U.K., Bhattacharya A., Nag A. (2019). Functionalized Chitosan-Carbon Dots: A Fluorescent Probe for Detecting Trace Amount of Water in Organic Solvents. ACS Omega.

[54] Tian H., Dai Y., Fu W., Liu H., Li M., Lv M., Yin X. (2020). Dansyl-Modified Carbon Dots with Dual-Emission for PH Sensing, Fe^3+^ Ion Detection and Fluorescent Ink. RSC Adv..

[55] Abdelhamid H.N., Wu H.F. (2018). Selective Biosensing of Staphylococcus Aureus Using Chitosan Quantum Dots. Spectrochim. Acta Part A Mol. Biomol. Spectrosc..

[56] Abdelhamid H.N., El-Bery H.M., Metwally A.A., Elshazly M., Hathout R.M. (2019). Synthesis of CdS-Modified Chitosan Quantum Dots for the Drug Delivery of Sesamol. Carbohydr. Polym..

[57] Song J., Zhao L., Wang Y., Xue Y., Deng Y., Zhao X., Li Q. (2018). Carbon Quantum Dots Prepared with Chitosan for Synthesis of CQDs/AuNPs for Iodine Ions Detection. Nanomaterials.

[58] Yang Y., Cui J., Zheng M., Hu C., Tan S., Xiao Y., Yang Q., Liu Y. (2011). One-Step Synthesis of Amino-Functionalized Fluorescent Carbon Nanoparticles by Hydrothermal Carbonization of Chitosan. Chem. Commun..

[59] Zhan J., Peng R., Wei S., Chen J., Peng X., Xiao B. (2019). Ethanol-Precipitation-Assisted Highly Efficient Synthesis of Nitrogen-Doped Carbon Quantum Dots from Chitosan. ACS Omega.

[60] Rafiee F., Tajfar N., Mohammadnejad M. (2021). The Synthesis and Efficiency Investigation of a Boronic Acid-Modified Magnetic Chitosan Quantum Dot Nanocomposite in the Detection of Cu^2+^ Ions. Int. J. Biol. Macromol..

[61] Chagas J.A.O., Crispim G.O., Pinto B.P., San Gil R.A.S., Mota C.J.A. (2020). Synthesis, Characterization, and CO_2_ Uptake of Adsorbents Prepared by Hydrothermal Carbonization of Chitosan. ACS Omega.

[62] Liang Z., Kang M., Payne G.F., Wang X., Sun R. (2016). Probing Energy and Electron Transfer Mechanisms in Fluorescence Quenching of Biomass Carbon Quantum Dots. ACS Appl. Mater. Interfaces.

[63] Keerthana A.K., Ashraf P.M. (2020). Carbon Nanodots Synthesized from Chitosan and Its Application as a Corrosion Inhibitor in Boat-Building Carbon Steel BIS2062. Appl. Nanosci..

[64] Janus Ł., Piatkowski˛ M., Radwan-Pragłowska J., Bogdał D., Matysek D. (2019). Chitosan-Based Carbon Quantum Dots for Biomedical Applications: Synthesis and Characterization. Nanomaterials.

[65] Lu W., Gao Y., Jiao Y., Shuang S., Li C., Dong C. (2017). Carbon Nano-Dots as a Fluorescent and Colorimetric Dual-Readout Probe for the Detection of Arginine and Cu^2+^ and Its Logic Gate Operation. Nanoscale.

[66] Jiang Q., Jing Y., Ni Y., Gao R., Zhou P. (2020). Potentiality of Carbon Quantum Dots Derived from Chitin as a Fluorescent Sensor for Detection of ClO^−^. Microchem. J..

[67] Xiao D., Yuan D., He H., Lu J. (2013). Microwave-Assisted One-Step Green Synthesis of Amino-Functionalized Fluorescent Carbon Nitride Dots from Chitosan. Luminescence.

[68] Zhao L., Wang Y., Zhao X., Deng Y., Xia Y. (2019). Facile Synthesis of Nitrogen-Doped Carbon Quantum Dots with Chitosan for Fluorescent Detection of Fe^3+^. Polymers.

[69] Chandra S., Pathan S.H., Mitra S., Modha B.H., Goswami A., Pramanik P. (2012). Tuning of Photoluminescence on Different Surface Functionalized Carbon Quantum Dots. RSC Adv..

[70] Feng Z., Hakkarainen M., Grützmacher H., Chiappone A., Sangermano M. (2019). Photocrosslinked Chitosan Hydrogels Reinforced with Chitosan-Derived Nano-Graphene Oxide. Macromol. Chem. Phys..

[71] Wu Q., Zhang S., Li S., Yan Y., Yu S., Zhao R., Huang L. (2022). Chitosan-Based Carbon Dots with Multi-Color-Emissive Tunable Fluorescence and Visible Light Catalytic Enhancement Properties. Nano Res..

[72] Riahi Z., Rhim J.W., Bagheri R., Pircheraghi G., Lotfali E. (2022). Carboxymethyl Cellulose-Based Functional Film Integrated with Chitosan-Based Carbon Quantum Dots for Active Food Packaging Applications. Prog. Org. Coat..

[73] Lin X., Xiong M., Zhang J., He C., Ma X., Zhang H., Kuang Y., Yang M., Huang Q. (2021). Carbon Dots Based on Natural Resources: Synthesis and Applications in Sensors. Microchem. J..

[74] Kang C., Huang Y., Yang H., Yan X.F., Chen Z.P. (2020). A Review of Carbon Dots Produced from Biomass Wastes. Nanomaterials.

[75] Feng Y., Li R., Zhou P., Duan C. (2022). Non-Toxic Carbon Dots Fluorescence Sensor Based on Chitosan for Sensitive and Selective Detection of Cr (VI) in Water. Microchem. J..

[76] Atchudan R., Jebakumar Immanuel Edison T.N., Shanmugam M., Perumal S., Somanathan T., Lee Y.R. (2021). Sustainable Synthesis of Carbon Quantum Dots from Banana Peel Waste Using Hydrothermal Process for in Vivo Bioimaging. Phys. E Low-Dimens. Syst. Nanostruct..

[77] Xu J., Qi Q., Sun L., Guo X., Zhang H., Zhao X. (2022). Green Fluorescent Carbon Dots from Chitosan as Selective and Sensitive “off-on” Probes for Nitrite and “on-off-on” Probes for Enrofloxacin Detection. J. Alloys Compd..

[78] Liu G., Li B., Liu Y., Feng Y., Jia D., Zhou Y. (2019). Rapid and High Yield Synthesis of Carbon Dots with Chelating Ability Derived from Acrylamide/Chitosan for Selective Detection of Ferrous Ions. Appl. Surf. Sci..

[79] Perumal S., Atchudan R., Edison T.N.J.I., Lee Y.R. (2021). Sustainable Synthesis of Multifunctional Carbon Dots Using Biomass and Their Applications: A Mini-Review. J. Environ. Chem. Eng..

[80] Horo H., Saha M., Das H., Mandal B., Kundu L.M. (2022). Synthesis of Highly Fluorescent, Amine-Functionalized Carbon Dots from Biotin-Modified Chitosan and Silk-Fibroin Blend for Target-Specific Delivery of Antitumor Agents. Carbohydr. Polym..

[81] Liu X., Pang J., Xu F., Zhang X. (2016). Simple Approach to Synthesize Amino-Functionalized Carbon Dots by Carbonization of Chitosan. Sci. Rep..

[82] Trung L.G., Subedi S., Dahal B., Truong P.L., Gwag J.S., Tran N.T., Nguyen M.K. (2022). Highly Efficient Fluorescent Probes from Chitosan-Based Amino-Functional Carbon Dots for the Selective Detection of Cu^2+^ Traces. Mater. Chem. Phys..

[83] Pan T., Chen H., Gao X., Wu Z., Ye Y., Shen Y. (2022). Engineering Efficient Artificial Nanozyme Based on Chitosan Grafted Fe-Doped-Carbon Dots for Bacteria Biofilm Eradication. J. Hazard. Mater..

[84] Xu J., Wang Y., Sun L., Qi Q., Zhao X. (2021). Chitosan and κ-Carrageenan-Derived Nitrogen and Sulfur Co-Doped Carbon Dots “on-off-on” Fluorescent Probe for Sequential Detection of Fe^3+^ and Ascorbic Acid. Int. J. Biol. Macromol..

[85] Chen Y., Zhao C., Wang Y., Rao H., Lu Z., Lu C., Shan Z., Ren B., Wu W., Wang X. (2020). Green and High-Yield Synthesis of Carbon Dots for Ratiometric Fluorescent Determination of PH and Enzyme Reactions. Mater. Sci. Eng. C.

[86] Lv X., Man H., Dong L., Huang J., Wang X. (2020). Preparation of Highly Crystalline Nitrogen-Doped Carbon Dots and Their Application in Sequential Fluorescent Detection of Fe^3+^ and Ascorbic Acid. Food Chem..

[87] Shen L., Chen M., Hu L., Chen X., Wang J. (2013). Growth and Stabilization of Silver Nanoparticles on Carbon Dots and Sensing Application. Langmuir.

[88] Younis M.R., He G., Lin J., Huang P. (2020). Recent Advances on Graphene Quantum Dots for Bioimaging Applications. Front. Chem..

[89] Del Carpio-Perochena A., Bramante C.M., Duarte M.A.H., Moura M.R.d., Aouada F.A., Kishen A. (2015). Chelating and Antibacterial Properties of Chitosan Nanoparticles on Dentin. Restor. Dent. Endod..

[90] Fan K., Zhang M., Guo C., Dan W., Devahastin S. (2021). Laser-Induced Microporous Modified Atmosphere Packaging and Chitosan Carbon-Dot Coating as a Novel Combined Preservation Method for Fresh-Cut Cucumber. Food Bioprocess Technol..

